# An all-ultrasound cranial imaging method to establish the relationship between cranial FUS incidence angle and transcranial attenuation in non-human primates in 3D

**DOI:** 10.21203/rs.3.rs-3017935/v1

**Published:** 2023-06-13

**Authors:** Aparna Singh, Sergio Jiménez-Gambín, Elisa E Konofagou

**Affiliations:** 1. Department of Biomedical Engineering, Columbia University, New York, NY, USA; 2. Department of Radiology, Columbia University, New York, NY, USA

## Abstract

Focused ultrasound (FUS) is a non-invasive and non-ionizing technique which deploys ultrasound waves to induce bio-effects. When paired with acoustically active particles such as microbubbles (MBs), it can open the blood brain barrier (BBB) to facilitate drug delivery inhibited due to the presence of BBB. One of the parameters that affects the FUS beam propagation is the beam incidence angle on the skull. Prior work by our group has shown that, as incidence angles deviate from 90°, FUS focal pressures attenuate and result to a smaller BBB opening volume. The incidence angles calculated in our prior studies were in 2D and used skull information from CT. The study presented herein develops methods to calculate incidence angle in 3D in non-human primate (NHP) skull fragments using harmonic ultrasound imaging without using ionizing radiation. Our results show that ultrasound harmonic imaging is capable of accurately depicting features such as sutures and eye-sockets of the skull. Furthermore, we were able to reproduce previously reported relationships between the incidence angle and FUS beam attenuation. We also show feasibility of performing ultrasound harmonic imaging in *in-vivo* non-human primates. The all-ultrasound method presented herein combined with our neuronavigation system stands to increase more widespread adoption of FUS and render it accessible by eliminating the need for CT cranial mapping.

## Introduction

In neurological diseases such as epilepsy, Parkinson’s, and Alzheimer’s, the first line of treatment is typically pharmacological. These medications, however, have not been shown efficacious over time due to developed tolerance^10^ and progression of disease. In those cases, brain stimulation methodologies, such as deep brain stimulation (DBS) can treat the symptoms especially in advanced stages of Parkinson’s disease where in one randomized trial, DBS of subthalamic nucleus caused greater improvements from baseline to six months in quality of life in Parkinson’s disease patients when compared to medication alone^10^. However, DBS has resulted in serious adverse events in 13 percent of the cases including a fatal intracerebral hemorrhage, possibly due to it being invasive in nature^12^. Another widely used brain stimulation method, transcranial magnetic stimulation (TMS), has shown reduction in seizures in patients with epilepsy with longer stimulation groups reporting fewer seizures than shorter stimulation groups^19^. However, TMS suffers from poor depth penetration and limited spatial resolution^2,43^.

Focused ultrasound (FUS) is an exciting, relatively new alternative technique as it is not only inherently non-invasive, but also achieves greater depth penetration. Several clinical trials have shown the potential of FUS for treating essential tremors^13,26^, for providing pain relief^5^ and, for blood-brain barrier (BBB) opening applications^1,29^. For all FUS-guided therapies, the gold standard method for targeting is MRI^1,6,13,26^. MRI provides excellent tissue contrast with tissue temperature monitoring capabilities^42^ and helps with predicting therapeutic outcomes such as FUS ablation. However, it fails to monitor microbubble activity, which is paramount to safe and successful FUS BBB opening procedures. A second imaging modality that is widely used for FUS pre-planning is CT as it provides with important acoustic parameters of the skull and brain that are essential to predicting FUS beam path, attenuation of FUS pressures due to thickness and density of skull and predicting the incidence angle of FUS beams onto the skull^20,21,45^. However, CT is costly, ionizing, and without intra-monitoring capabilities.

Another alternative for guidance and particle activity monitoring is ultrasound. An ultrasound imaging array, when set on receive mode, can monitor this microbubble activity at high frame rates and inform us of safety of neuromodulation procedures or blood-brain barrier (BBB) opening. Past research published by our group has developed methods to detect microbubble activity during BBB opening^39,40^. Other significant developments have been reported over the past few years where ultrasound imaging arrays have been used to identify anatomical sutures on mice skull and has been used to guide blood-brain barrier opening in small animals and deliver molecules across the BBB^7,8^. Additionally, advancements in B-mode ^20^image processing have made it possible for ultrasound imaging transducers to be used for brain vascular imaging^11,14^, perform transcranial imaging through the human skull^31^, and for detecting functional activity in brain in small animals^3,28^ and in newborns^9^. Overall, these recent developments of ultrasound-guided focused ultrasound technologies enabled the use of transcranial power Doppler image to guide BBB opening in rats^36^. Ultrasound monitoring has also played a huge role in other neuromodulation procedures that do not involve BBB opening. In one study by our group^25^, researchers showed that during neuromodulation of peripheral nervous system (PNS), ultrasound imaging transducer can be used to image real time displacement and cavitation, thereby informing us about the intricate interplay of cavitation and displacement in causing neuromodulation of PNS. In addition, other groups have also shown that ultrasound can be used to detect drug release from nanoparticles after FUS application^24^. Thus, ultrasound imaging guidance and monitoring can provide an efficient, reliable and promising alternative for FUS applications. In this study, we aimed to further explore the capability of real-time ultrasound harmonic B-Mode images for FUS applications. Studies published by our group has shown that incidence angle, angle between the normal vector to skull plane and the transducer plane, is critical for the reliable and reproducible BBB opening in non-human primates^20^and humans^4,22,23^. In this study, we developed methods to use an all-ultrasound device which can perform cranial imaging and predict FUS incidence angles on the skull using ultrasound imaging. We validated this technique in two *ex-vivo* NHP skulls and showed the feasibility of transcranial imaging in two *in-vivo* NHP experiments.

## Results:

### K-wave simulation predictions:

**Fig 1:**
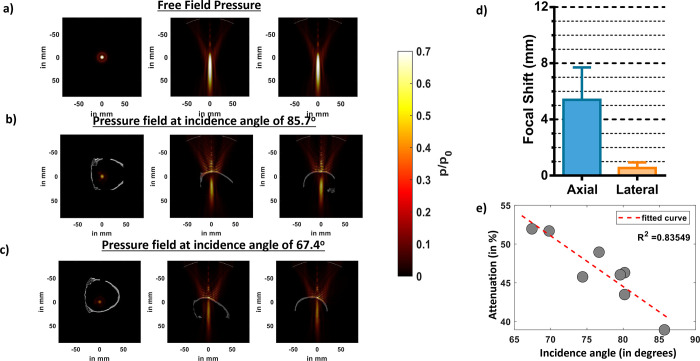
Effects of different FUS incidence angles on ex-vivo skull predicted by k-wave simulations. a) Lateral and axial pressure fields distributions, respectively, in free field propagations. b) Lateral and axial pressure fields distributions in presence of skull at the best incidence angle of 85.7 degree. c) Lateral and axial pressure fields distributions at an incidence angle of 67.4 degree. d) Average axial and lateral focal shift computed from pressure fields in presence of skull at all incidence angles. e) Graph showing a trend of increased attenuation away from normal incidence angle (90 degrees).

**Fig 2:**
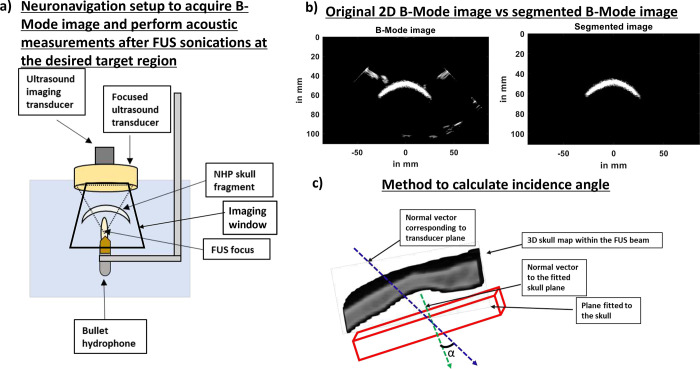
Transcranial skull imaging using ultrasound imaging transducer P4–2. a) NHP skull fragment B-Mode images were acquired using P4–2 by performing raster scan of the entire skull. b) B-Mode image (left) was segmented (right) below to only contain the skull. c) Angle between normal vector fitted to the skull plane and normal vector corresponding to transducer plane was used to calculate incidence angle.

Before performing k-wave simulations, we rotated the skull using the ‘imrotate3’ function of MATLAB. We, then, calculated the incidence angle by computing the angle between the normal vector fitted on to the skull plane and the normal vector corresponding to the transducer plane (Fig 2b). The results of k-Wave simulations (Fig 1) show that the presence of skull at different incidence angles attenuate pressure fields where the pressure attenuated by as much as 47% when the incidence was 85.7° in Fig 1b. The attenuation further increased to up to 60% when incidence angle was 67.4° in Fig 1c. We then compared how attenuation was affected at different FUS incidence angles to the skull in Fig 1f. We observed that as the incidence angle approximated to 90 degrees, the attenuation decreased and that there was a strong correlation (R^2^ = 0.84) between the attenuation and FUS incidence angle. We then used all our eight simulations and compared the focal shift with respect to the free field in Fig 1d and found that the average axial and lateral focal shift was 5.0±2.4mm and 0.38±0.27mm respectively.

### 3-D reconstruction of the cranial NHP skull map:

**Fig 3.**
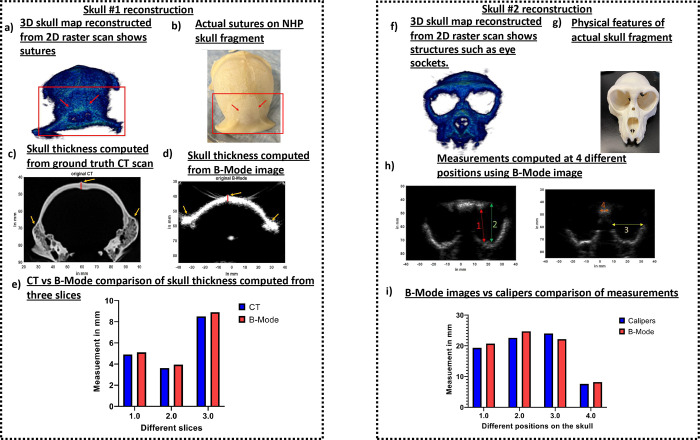
a) 2D raster scans of skull #1 helped reconstruct 3D skull map. This 3D skull map shows sutures. b) The sutures visible on 3D skull map were also present on the physical skull. c) Skull thickness was computed using CT of the skull. Red line shows the region that was measured. d) B-Mode image of the skull consisted of the same anatomical region. Red line denotes the region where measurements were computed from. e) Skull thickness was computed from 3 different CT and B-Mode slices where anatomical regions could be matched. Comparing skull thickness values evaluated from CT and B-Mode show that the values between them were comparable. f) 2D raster scan of skull #2 helped reconstruct its 3D skull map. We can see the eye sockets along with other prominent structures. g) Physical skull also shows prominent features present on the 3D skull map. h) A total of 4 measurements were taken to compute dimensions of eye sockets and its neighboring areas using its B-Mode image. i) The B-Mode computed measurements were then compared to physical caliper measurements. These measurements were found to be comparable.

To generate a skull map, we manually segmented the skull from each of the 500 reconstructed, harmonic B-Mode images with a resolution of 0.14mm × 0.14mm × 0.2mm (Fig 2b). We then assembled each of those planes to generate a skull mask using the volshow function of MATLAB. The 3D skull map reconstructed from 2D raster scans for skull number 1 in Fig 3a shows the skull sutures identified using the red arrows. These sutures are also present in the physical skull in Fig 3b. We then identified similar slices in CT (Fig 3c) and reconstructed harmonic B-Mode (Fig 3d) via comparing features (in yellow arrows) present in both CT and B-Mode slices. Thereafter, we calculated the thickness of the bone in those slices. We compared bone thickness in a total of 3 slices in Fig 3e and found that the thickness computation from B-Mode and CT slices were comparable. In slice 1, the measurements computed from CT vs B-mode were equal to 4.9mm vs 5.1mm, in slice 2 it was 3.62mm vs 3.95mm, and in slice 3 was 8.5mm vs 8.9mm. The 3D skull map for NHP skull # 2 in Fig 3f clearly shows eye sockets and features that are present in the orthogonal skull in Fig 3g. We compared the physical measurements of the eye sockets computed via calipers (Mitutoyo, Aurora, IL) with that of computed from B-Mode at 4 different positions shown via different color arrows in Fig 3h. We found that the measurements, of calipers vs B-Mode, for location 1 was 19.34mm vs 20.7mm, for location 2 was 22.57mm vs 24.7mm, for location 3 was 24mm vs 22.17mm, and for location 4 was 7.6mm vs 8.17mm.

### *Ex-vivo* incidence angle estimation using harmonic B-Mode imaging and FUS pressure attenuation:

**Fig 4.**
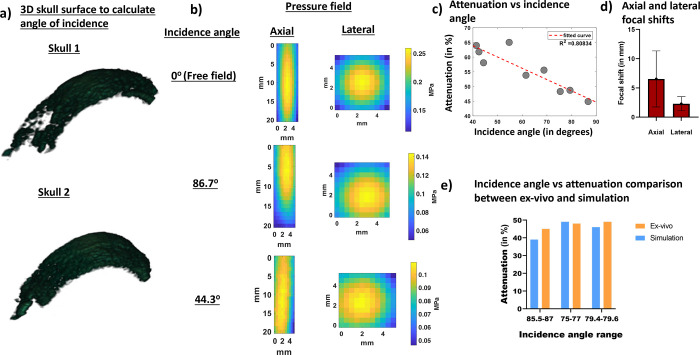
a) Example of a 3D surface of the skull that was used to calculate incidence angle for both skulls. A total of 9 3D surfaces were used to calculate incidence and angle and establish its relationship with attenuation. b) Pressure fields recorded in free field and transcranially at the best and worst incidence angle show a reduction in attenuation as incidence angle gets further away from 90°. c) Graph that shows relationship between incidence angles, calculated from 3D B-mode images, and attenuation calculated from pressure fields. d) Focal shifts evaluated from pressure field maps recorded using hydrophone at different incidence angles. e) Graph comparing similar incidence angles between ex-vivo and simulations.

To calculate the incidence angle through two ex-vivo NHP skulls, we acquired and segmented 70 slices of harmonic B-Mode images, each 0.5mm apart using the imaging transducer that was co-axially aligned with our FUS transducer. Our imaging sequence comprised 256 diverging waves acquired at 2-MHz with phased array (P4–2, ATL, Philips). B-mode images were acquired at a depth of 110mm. To obtain a single harmonic B-Mode image, a 2-cycle diverging wave at 2MHz followed by another 2-cycle diverging wave with opposite polarity was delivered. The final reconstructed B-Mode image was then saved onto the computer and the transducer was then moved to the next plane 0.5mm away to ultimately acquire 70 slices. We used this partial 3D skull map in Fig. 4a for both skulls to estimate FUS incidence angle on the skull (Fig 2c). We then performed FUS sonications with our H-231 FUS single-element transducer (Sonic Concepts, Bothell, WA). Our FUS transducer (F0 = 250 kHz, OD = 110mm, ID = 44mm, and focal distance = 110mm), was coaxially aligned with the imaging transducer. FUS pulses of 15 cycles at a PRF of 100 Hz at 0.3 MPa were transmitted thereafter and we used bullet hydrophone (H0400, ONDA Corporation, Sunnyvale, CA) to record field pressures underneath the skull. For skull #1 in Fig 4b, we observe that the FUS incidence angle on the skull impacts the intensity of transcranial FUS pressure field recorded via hydrophone. As a result, similar to what was predicted in the simulation an incidence angle of 86.7 degrees shows lower attenuation than incidence angle of 44.3 degrees. When attenuation results are combined for both skulls in Fig 4c, the same linear relationship as that of the simulation is obtained whereas the incidence angle approached 90 degrees, the attenuation decreased. The dependence of attenuation on the incidence angle was found to be high (R^2^ = 0.81). We also calculated average axial and lateral focal shifts in Fig 4d from the beam path generated and found an average axial focal shift of 6.54±4.81mm and lateral focal shift of 2.31±1.19mm. When examining incidence angles vs attenuation, there were 3 incidence angles in Fig 4e that were comparable between simulation and experimental condition. An incidence angle of 85.7 degrees in simulation resulted in 39% attenuation whereas an incidence of 86.7 degrees in experimental condition resulted in 45% attenuation. Similarly, an incidence angle of 76.7 degrees in simulation resulted in 49% attenuation whereas an incidence of 75.4 degrees in experimental condition resulted in 48% attenuation. Finally, an incidence angle of 79.6 degrees in simulation resulted in 46% attenuation whereas an incidence of 79.4 degrees in experimental condition resulted in 49% attenuation.

### *In-vivo* harmonic cranial B-Mode imaging:

**Fig 5.**
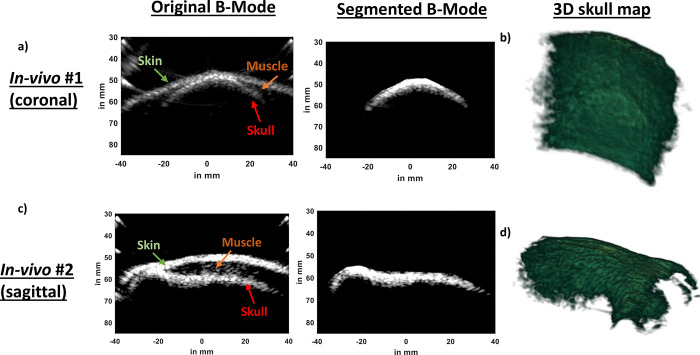
a) A coronal slice of *In-vivo* B-Mode scan shows the skin, muscle, and skull. We took multiple B-Mode coronal slices of our first in-vivo NHP and segmented the skull from each of those B-Mode images b) We reconstructed a 3D skull map using multiple coronal slices which shows sutures visible on the skull surface. c) A sagittal slice of our second in-vivo study also reveals the skin, muscle, and skull. We segmented the skull from the B-Mode image. d) After segmenting the skull out from multiple sagittal slices of our second in-vivo study, we reconstructed 3D skull map which shows eye sockets.

We performed harmonic cranial B-Mode imaging in two *in-vivo* NHPs (Rhesus Macaques, Male, 8 years old) using our ultrasound guided FUS setup driven by Robotic Arm (Universal Robots, UR5E). In our first NHP imaging study, we collected coronal slices. In the original B-Mode image in Fig 5a, we can identify skin, muscle, and skull. We manually segmented out the skull from original B-Mode image (Fig 5a) and created 3D skull map in Fig 5b. In the 3D skull map rendered using the ‘volshow’ function of MATLAB, sutures inside the skull surface are depicted which is a characteristic of NHP skull. In our second in-vivo setting in Fig 5c, we performed imaging in the sagittal plane. Similar to our first in-vivo imaging case, we identified skin, muscle, and skull layers. We manually segmented out the skull and assembled all 500 slices to generate a 3D skull map in Fig 5d. In the sagittal plane, we were able to recover eye sockets.

## Discussion:

FUS is a non-invasive and non-ionizing therapeutic technology that can treat neurological conditions by focusing ultrasound waves at desired target regions. Due to its non-invasive nature, it has been FDA approved for ablation for uterine fibroids and essential tremors. The clinical method for targeting FUS is using MRI thermometry^26,32,46^. To target accurately and calculate FUS attenuation for transcranial applications, CT images of the skull are utilized^18,27^. Such targeting methods have been used towards blood brain barrier opening and neuromodulation procedures. However, using MRI and CT collectively for targeting and trajectory planning can render FUS ionizing and less accessible. In this study, we demonstrated the feasibility of using transcranial ultrasound B-Mode imaging to compute the incidence angle during FUS for BBB opening in NHP using a clinical system^35^. Using an all-ultrasound system is advantageous when compared to other modalities such as MRI and CT. In addition to providing real-time guidance, ultrasound also provides tools for real-time monitoring thereby, enabling performing repeated FUS procedures reliably and cost-efficiently. Our neuronavigation clinical system, which has been used in this study, has successfully performed BBB opening and by developing methods to image skulls and accurately predict FUS incidence angles, we can enhance the applicability of our clinical system for FUS procedures. We showed that we can successfully use this clinical system to achieve our desired objective and that our experimental results are comparable to simulations Our simulation results showed a determinant coefficient of 0.85 between FUS angle incidence and attenuation. This is in line with published studies where a similar relationship and determinant coefficient was seen between volume of BBB opening and incidence angle at fixed input pressures ^20,21^.

### Cranial harmonic images of the skull accurately depicted skull landmarks:

Cranial B-Mode images of *ex-vivo* skulls in Fig 3 were able delineate skull boundaries. This enabled segmentation of the skull and eventually helped with 3D skull map reconstruction. The 3D skull map was able to show sutures and eye sockets present on the skull. Furthermore, B-Mode images were able correctly infer the skull thickness and eye socket width measurements. These will be unprecedentedly advantageous for targeting purposes as these anatomical sutures and locations can be further used with our neuronavigation system for real-time registration and FUS guidance^4,22,34,35,44^.

### Comparison between simulation and experimental findings:

Our *ex-vivo* pressure measurements were found to be in good agreement with simulations. A determinant coefficient of 0.81 was observed in experimental condition which was in line with our simulations. *Ex-vivo* pressure attenuation measurements closely resembled simulation pressure attenuation. When comparing attenuation values between simulation and physical measurements, the average error was within 6%. Furthermore, the average axial focal shifts were similar in magnitude when experimental conditions were compared to simulations. However, the lateral focal shift was found to be 2mm greater in experiments than in simulations. The average lateral shift in both cases was calculated by averaging contributions from all incidence angles. In experimental conditions, some of the incidence angles were lower than 45 degrees. On the other hand, in the simulations, lowest incidence angle was 69.8 degrees. A wider of incidence angle may have, thus, contributed to larger average lateral shift the in experimental condition.

### In-vivo harmonic imaging revealed certain features on the NHP skull:

We further showed feasibility of acquiring harmonic B-Mode images in two different in-vivo cases. In both cases in Fig 5, the skin, muscle, and skull layers could be clearly distinguished. Furthermore, after segmentation and reconstruction of skull map, we were able to identify sutures and key anatomical landmarks such as eye sockets. These features will enable us to use harmonic B-Mode imaging derived landmarks to guide and open the BBB using our clinical system in NHP.

While our method can perform cranial harmonic B-Mode imaging and predict the angle of incidence in transcranial FUS applications, it has some limitations. This method relies on slice-by-slice segmentation which can present some challenges. Manual segmentation can be time consuming and subjective and need to be done off-line. Thus, to render this procedure clinically relevant, segmentation needs to be performed automatically with real-time feedback such as through the use of machine learning approaches.

### Conclusion

In this study, we developed cranial harmonic B-Mode imaging method to predict angle of incidence for FUS therapies, thereby eliminating the need to use ionizing methods, such as CT, for targeting purposes. We, first, performed cranial imaging using our clinical neuronavigation system, with ultrasound imaging transducer coaxially aligned with FUS transducer, on ex-vivo skulls. We used these cranial images to predict FUS beam incidence angle on the skull and compared it FUS pressure field recorded, via bullet hydrophone, at those respective incidence angles. The results showed a decreasing trend in attenuation as incidence angle approximated to normal. This agreed with our k-wave simulation results and previously published results Click or tap here to enter text. We, then, showed feasibility of performing harmonic cranial imaging in in-vivo NHPs. Our future work will incorporate in-vivo harmonic cranial imaging for targeting blood brain-barrier opening in large animals and clinical studies and will be used to calculate FUS incidence angles during brain therapies in real-time.

## Materials and Methods:

### Simulations using k-Wave:

Numerical simulations to predict focused ultrasound pressure through NHP skull were modelled using k-Wave package ^37,38^. This method is selected as it provides low numerical dispersion as compared with finite-differences methods^15^.

First, an ex-vivo NHP skull was degassed for 24 hours. Then, a CT scan of the skull fragment was acquired using a clinical CT scanner (Siemens BioGraph mCT 64 Slices Scanner, Siemens Healthcare), with a resolution of 0.24 × 0.24 × 0.6 mm^3^. The CT data was converted from Hounsfield Units to heterogeneous acoustic properties of sound speed and density using the linear-piecewise polynomials proposed previously^30,41^. The absorption of the NHP skull is assumed to be heterogenous where the maximum absorption was 0.68 dB/cm at 250 kHz with a frequency-power law exponent of two^33^. We used an isotropic grid with a spatial step of 0.4 mm, which corresponds to a spatial sampling of 15 points per wavelength (ppw) in water at the working frequency. Even though the simulation’s convergence, stability and accuracy can be reached working at 5–6 ppw in k-Wave^16,17^, we used 15 ppw to have a minimum of 8 grid points across the NHP skull thickness to capture microstructure and irregularities of the skull. The numerical temporal step was set to 26.7 ns and 54 ns for the simulations with and without the NHP skull, respectively, leading to a Courant-Friedrichs-Lewy number of 0.2 in both cases.

The H-231 FUS single-element transducer (Sonic Concepts, Bothell, WA) was then modelled in k-Wave. A bowl was modelled with dimensions which were comparable to the actual transducer (f_0_ = 250 kHz, outer diameter (OD) = 110 mm, inner diameter (ID) = 44 mm, and focal distance = 110 mm). The geometric focus was placed 3 cm below the skull surface. The skull was rotated using imrotate3 MATLAB function such that it created different incidence angles. We, then, performed a GPU-accelerated 3D acoustic k-Wave simulation on a workstation PC (Dell) with a NVIDIA Quadro P6000 GPU (Nvidia, Santa Clara, CA). The pixel resolution was 0.4 × 0.4 × 0.4 mm3 with a 3D grid composed of total of 271 × 215 × 215 voxels. The CT data was resampled to fit the k-Wave simulation voxel size. The maximum pressure was recorded for every voxel in the simulation grid. A total of 8 simulations were performed at 8 different incidence angles. Additionally, another simulation was performed to mimic free-field. Resulting pressure fields were used to obtain values of focal shift, focus full width at half maximum (FWHM), and skull insertion loss. The relationship between attenuation and incidence angle was then established.

### Cranial B-Mode imaging:

To obtain 3D transcranial skull map, B-mode images of two ex-vivo skulls were acquired at 2MHz with phased array (P4–2, ATL, Philips) using 256 diverging waves. B-mode images were acquired at a depth of 110mm. To obtain a single B-Mode image, a 2-cycle diverging wave at 2MHz followed by another 2-cycle diverging wave with opposite polarity was delivered to perform harmonic imaging. After acquisition of a B-Mode image of a plane, imaging transducer was moved 0.2mm to acquire image of the next plane. A total of 500 planes were acquired to reconstruct image of an entire skull. This process was repeated for the second skull. After obtaining B-Mode image of each plane, skull was segmented out using ‘roipoly’ function of MATLAB (Fig 2b). After segmenting skull from all planes, a 3D skull map was reconstructed. After raster scan, B-Mode slices of skull was compared to skull CT of skull number 1 to identify similar slices. Once identified, skull thickness from CT was compared to skull thickness obtained from B-Mode slices (Figs 3c and 3d). Skull thickness for skull number 1 was computed for 3 different slices. For skull number 2, calipers were used to measure distances at 4 different positions on the physical skull (Fig 3h). The measurements from calipers were compared to measurements extracted from B-Mode. Our initial 3D scan was aimed at reconstructing the whole skull map.

### *Ex-vivo* setup for calculating incidence angle, performing FUS, and collecting pressure field maps:

For calculating incidence angle for FUS procedure, we acquired only 70 slices, 0.5mm apart using Robotic Arm (Universal Robots, UR5E). This helped us get 35mm of the NHP *ex-vivo* skull. We, then, moved the imaging transducer to centermost slice and sonicated the FUS transducer (F0 = 250 kHz, OD = 110mm, ID = 44mm, and focal distance = 110mm), which was coaxially aligned to the imaging transducer. FUS pulses of 15 cycles at a PRF of 100 Hz at 0.3 MPa were transmitted thereafter. The resulting pressure fields were recorded using bullet hydrophone (Fig 2a). A free field recording was also performed in absence of the skull and was compared to pressure fields obtained in the presence of skull. In order to create incidence angle, the skull was manually rotated. A total of 10 pressure fields, including one in free field, was obtained. After obtaining the pressure field and NHP *ex-vivo* B-Mode images, the images were segmented as previously mentioned. After segmenting the images, the 3D incidence angle was calculated between the central axis of the transducer (propagation direction) and the plane containing the sonicated skull surface (Fig 2c). We first obtained the grid points corresponding to the sonicated skull surface by evaluating the size of the beam covering the skull. Thereafter, we fitted the resulting surface to a plane. Finally, we calculated the incidence angle from the normal vector of this plane where incidence angle was computed to be 90°-α, where α is the angle between the normal vector of the skull fitted plane and the propagation direction vector of the transducer.

### *In-vivo* harmonic B-Mode imaging feasibility –

To show the feasibility of transcranial imaging in NHPs, we performed in-vivo imaging in two NHPs (Male rhesus-macaques, 8 years old). All procedures were reviewed and approved by the Columbia University Institutional Animal Care and Use Committee and performed in accordance with the relevant guidelines and regulations for animal research. Additionally, our study followed the ARRIVE guidelines. In the first NHP, we imaged coronal slices. For our second NHP, we imaged sagittal slices. We mounted our neuronavigation^4,34,35^ system onto a robotic arm (Universal Robots, UR5E). Once mounted on the robotic arm, 2 cycle diverging waves at 2MHz followed by 2 cycle diverging waves at 2 MHz with opposite polarity were delivered to acquire harmonic images of the skull. A total of 4 frames were acquired, saved, and then robotic arm was moved to another plane 0.5mm away from the previous plane. This procedure was repeated until 500 planes of data were acquired for each NHP. The planes were then segmented off-line (Fig 5b) and were assembled to create a 3D skull map of *in-vivo* NHPs.

## Data Availability

The dataset generated and used in this study will be available from the corresponding author upon request.
